# Supplementary material for: Mutations in PCYT2 disrupt etherlipid biosynthesis and cause a complex hereditary spastic paraplegia
Source: Brain. 2019 Oct 22;142(11):3382–97. doi: 10.1093/brain/awz291 (PMC6821184; doi:10.1093/brain/awz291)
Supplement: awz291_Supplementary_Data [file awz291_supplementary_data.zip › awz291-Suppl_data/Supplementary_Data4.zip!/Supplementary information 3 - fibroblast lipidomics.zip!/Fibroblast lipidomics/Controle vs Patient1/Statistical plots and data/PE[O] species/Volcano plot_alternative.pdf]

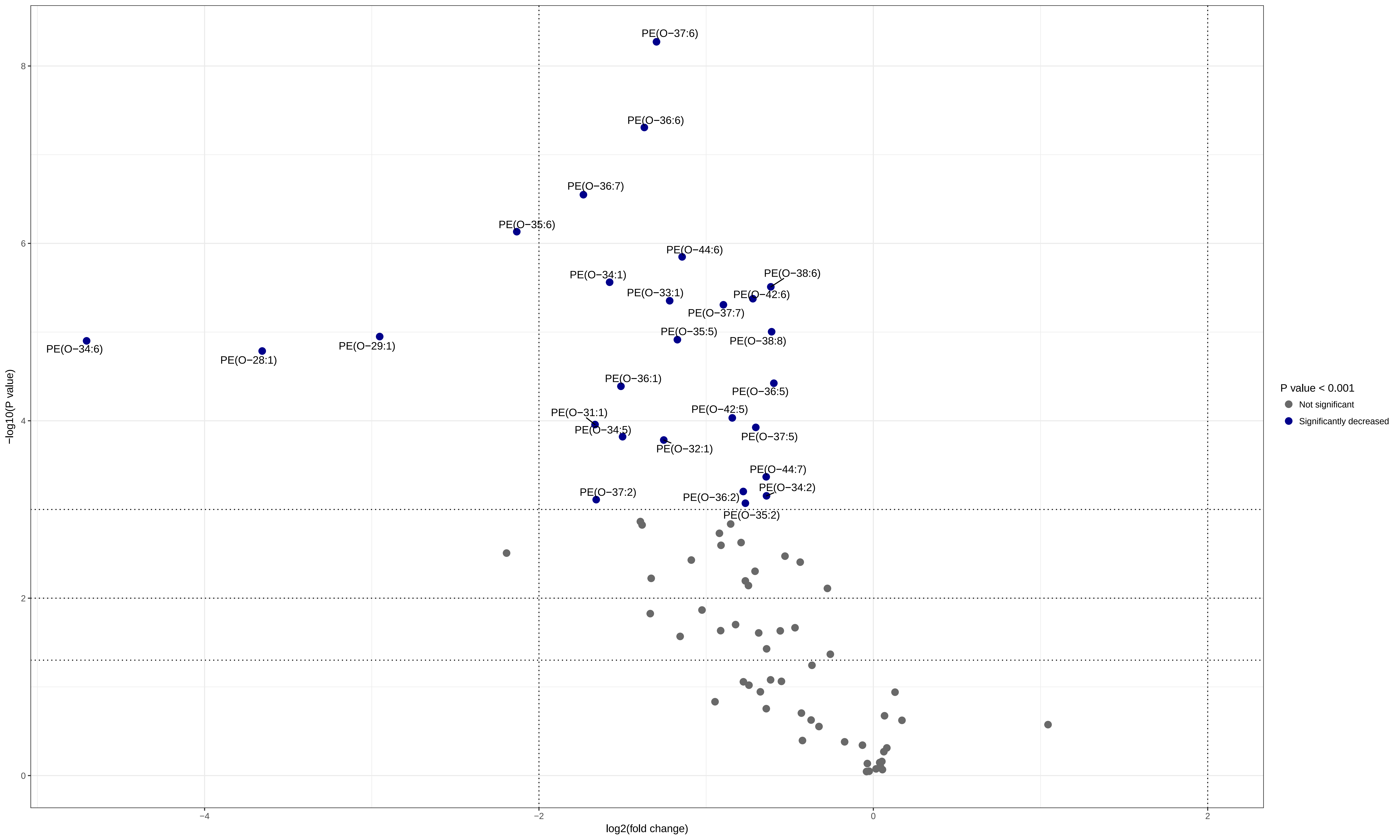
PE(O−37:6)
PE(O−36:6)
PE(O−36:7)
PE(O−35:6)
PE(O−44:6)
PE(O−34:1)
PE(O−38:6)
PE(O−33:1)
PE(O−42:6)
PE(O−37:7)
PE(O−35:5)
PE(O−38:8)
PE(O−34:6)
PE(O−28:1)
PE(O−29:1)
PE(O−36:1)
PE(O−36:5)
PE(O−31:1)
PE(O−42:5)
PE(O−34:5)
PE(O−37:5)
PE(O−32:1)
PE(O−44:7)
PE(O−34:2)
PE(O−37:2)
PE(O−36:2)
PE(O−35:2)
8
6
4
2
0
−4
−2
0
2
−log10(P value)
log2(fold change)
P value < 0.001
Not significant
Significantly decreased
